# Ecoepidemiological aspects of visceral leishmaniasis in an endemic area in the Steel Valley in Brazil: An ecological approach with spatial analysis

**DOI:** 10.1371/journal.pone.0206452

**Published:** 2018-10-30

**Authors:** Rosana S. Lana, Érika M. Michalsky, Lívia O. Lopes, Fabiana O. Lara-Silva, Jeiza L. Nascimento, Letícia C. Pinheiro, João C. França-Silva, Telma S. C. Mendes, Consuelo L. Fortes-Dias, Edelberto S. Dias

**Affiliations:** 1 Laboratório de Leishmanioses, Instituto René Rachou, Fundação Oswaldo Cruz, Belo Horizonte, Minas Gerais, Brazil; 2 Núcleo de Estudos em Saúde Pública e Envelhecimento, Instituto René Rachou, Fundação Oswaldo Cruz, Belo Horizonte, Minas Gerais, Brazil; 3 Laboratório de Leishmanioses, Instituto de Ciências Biológicas, Universidade Federal de Minas Gerais, Belo Horizonte, Minas Gerais, Brazil; 4 Departamento de Vigilância em Saúde, Secretaria Municipal de Saúde, Ipatinga, Minas Gerais, Brazil; 5 Laboratório de Enzimologia, Diretoria de Pesquisa e Desenvolvimento, Fundação Ezequiel Dias, Belo Horizonte, Minas Gerais, Brazil; Public Library of Science, UNITED KINGDOM

## Abstract

Leishmaniases are a group of infectious diseases transmitted by phlebotomine sand flies, and their distribution depends on the presence of vectors, parasites, reservoirs and susceptible hosts in the same environment. In the last decades, visceral leishmaniasis (VL) has become urbanized and reached economically important cities in countries within the transmission zone. Our study was conducted in one of those cities–Ipatinga–in the state of Minas Gerais, Brazil, where the first autochthonous case of VL dates back to 2011. Since no data regarding the epidemiological triad of VL (etiological agent/vector/domestic reservoir) were available for this city, we characterized the local entomological fauna, identified the presence of specific *Leishmania* DNA in the captured phlebotomine sand flies, and assessed the incidence of canine and human VL. For the entomological survey, we set twenty light traps in ten districts of the city with reports of human and canine VL. The insect captures were performed monthly, during one year, starting in March 2015. A total of 1501 specimens of phlebotomine sand flies belonging to 16 distinct species were captured, with predominance (61.9%) of *Lutzomyia longipalpis*. *Leishmania infantum* DNA was detected in *L*. *longipalpis* and in *Evandromyia cortelezzii* test samples. A total of 9,136 dogs were examined, 1,355 of which (14.8%) were serologically positive for VL. The cases were georeferenced and the data were plotted in thematic maps, along with human cases of VL registered by the local Department of Health, during the study period. Our results confirm that the VL transmission cycle is active in Ipatinga, with the presence of vectors carrying *Leishmania* DNA, canine and human cases of the disease. Spatial analysis allowed for the observation of a positive relationship between canine and human cases of VL and the identification of areas with high priority for control actions in the city. The mapping of high-risk areas, together with an epidemiological study in urban areas, is fundamental to improve the efficacy of the Program for Surveillance and Control of VL (PSCVL) in Brazil.

## Introduction

Leishmaniases are a group of infectious diseases caused by protozoa of the genus *Leishmania* Ross, 1903, and transmitted by the haematophagous Diptera from the family Psychodidae and subfamily Phlebotominae, the phlebotomine sand flies [1]. In the New World, two basic clinical forms of leishmaniases are described: tegumentary leishmaniasis (TL) and visceral leishmaniasis (VL). VL is the most severe form of the disease in humans and is fatal if left untreated.

In the American continent, eight countries are within the transmission zone of VL but most human cases (>95%) are reported in Brazil [2–4]. VL cases, as well as TL cases, are mandatorily reported to the Brazilian Ministry of Health through the National System of Notifiable Diseases (named SINAN). At first, VL characteristically occurred in rural environments, with a peridomiciliary profile. However, over the decades, it became urbanized due to several factors, such as deforestation, which reduced the availability of animals that served as a source of food for the vector, and the migratory process, which brought human and canine populations from rural areas, where the disease was endemic, to the periphery of cities [5–9].

The transmission chain of leishmaniases requires, in endemic areas, the concomitant presence of the vector, the parasite, the reservoir and the susceptible host. In the particular case of Brazil, the epidemiological triad of VL involves the parasite *Leishmania infantum* Nicolle, 1908 (syn. *Leishmania chagasi*) as the etiological agent, *Lutzomyia longipalpis* (Lutz & Neiva, 1912) phlebotomine sand flies as the main vector and dogs (*Canis familiaris*) as the principal domestic reservoirs [10–12]. Actually, it has been observed that canine VL usually precedes human cases of VL [13].

Historically, in 1953, the Brazilian government launched a campaign against VL due to the increasing number of cases in the country [14]. An official program, named Program for Surveillance and Control of Visceral Leishmaniasis (PSCVL), is still active and constitutes an integrated public health initiative specifically acting on all links in the epidemiological chain: diagnosis and treatment of human cases of VL as early as possible, control of the population of vectors by chemical spraying and removal of domestic canine reservoirs [15].

The actions of the PSCVL vary according to the local epidemiological transmission risk (ETR), which is based on the average number of reported human cases in the last three years (n) and varies from sporadic (n < 2.4) to medium (2.4 ≤ n < 4.4) or intense (n ≥ 4.4). The integrated control actions are generally intensified in areas with high or medium ETR (Brazilian Ministry of Health, 2009). In spite of these actions, VL has been expanding in Brazil, reaching highly urbanized cities including state capitals [16].

Currently, human VL is present in every State but Acre, in the five Brazilian geographic regions–named Center-west, North, Northeast, South and Southeast [17]. Between 2007 and 2016, 36,726 new cases of VL were notified to the Brazilian Ministry of Health, with an average incidence of 3,673 cases per year and 2,393 deaths in total. However, it is possible that the official data are underreported by 1.2 to 1.8 fold [2]. Thirteen per cent of the VL cases (totaling 4,771 cases) occurred in the state of Minas Gerais, which is located in the Southeast region of Brazil. And, in the course of time, VL spread from rural areas to urbanized and economically important cities in the state. This is the case of Ipatinga, a city located in the so-called Steel Valley, where large steel-producing companies—such as Usiminas and Arcelor Mittal–operate. The city also belongs to the Tourist Circuit of the Atlantic forest in Minas Gerais and offers trails, forests, ponds and waterfalls in its surrounds, therefore attracting a great number of tourists [18]. The last City Human Development Index (IDHM), published in 2010, was 0.771, which is considered high by the United Nations Development Program (UNDP), being the 220th highest in Brazil and the 16th highest in the State of Minas Gerais [19].

Ipatinga is considered an endemic area for TL with 286 cases reported to the SINAN of the Brazilian Ministry of Health, between 2007 and 2016. The first autochthonous case of VL was recorded in 2011. In the four following years, the number of cases of VL increased to eight, thirteen, 49, until it reached 51 in 2015 [17]. According to the criteria adopted by the Brazilian Ministry of Health, these numbers characterize a high local ETR [20]. Nonetheless, no data are available on the epidemiological triad for VL in the city, i.e. the parasite, the vector and the canine reservoir. Hence, in this study we characterized the local entomological fauna, we identified the presence of *Leishmania* in the captured phlebotomine sand flies, and we evaluated the prevalence of canine visceral leishmaniasis providing the first data on the VL epidemiological triad for Ipatinga.

## Material and methods

### Study area

**Fig 1:**
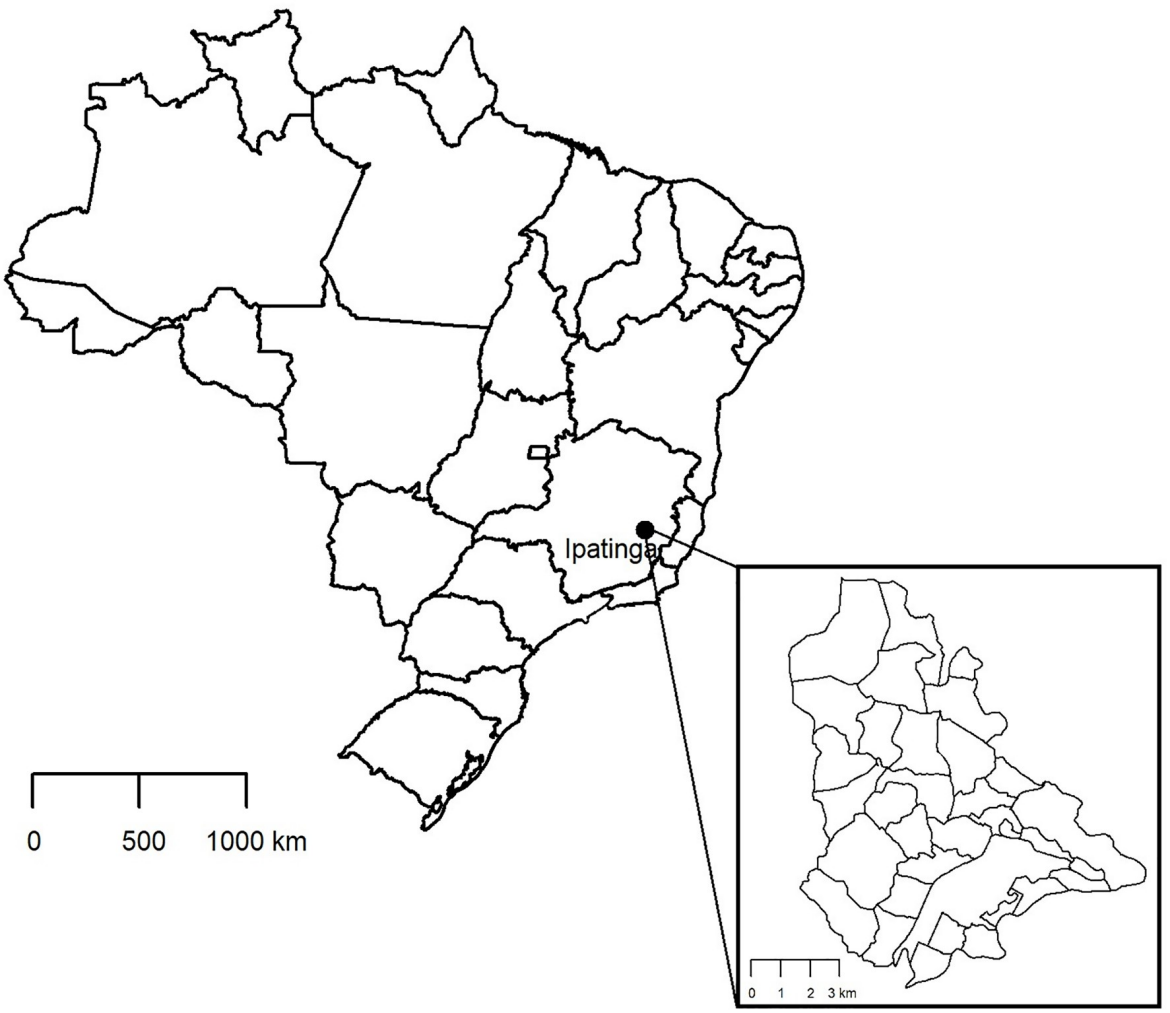
Geographic location of the city of Ipatinga in the Brazilian state of Minas Gerais. Ipatinga (black dot) is the main urban center in the region known as Steel Valley, due to the local settlement of large international steel-producing companies. Inset: Map of the city of Ipatinga with district subdivision.

The city of Ipatinga (19° 28' 06" S and 42° 32' 12" W) is located in the Southeast region of Brazil, in the East of the State of Minas Gerais (Fig 1). The city is approximately 209 km far from the state capital, Belo Horizonte (19° 55' 15" S and 43° 56' 16" W). The county of Ipatinga has an area of 164,884 km^2^, with 22,245 km^2^ in the urban perimeter. The estimated population is 239,468 inhabitants [21]; the climate of the region is warm and semi-humid tropical, according to the classification of Köppen.

The city is composed of 34 districts (Fig 1), ten of which were selected for our study: Bethânia, Bom Jardim, Canaã, Cariru, Cidade Nobre, Ideal, Iguaçu, Esperança, Veneza and Vila Celeste. All of them had VL cases notified to the local Health Department with the exception of the district of Cariru. Although no VL cases were reported before our study, this district accumulated 26 (33.8%) of the 77 cases of TL registered in the city, between 2012 and 2016 (data obtained from the Epidemiological Section of the local Department of Health, provided by T.S. Mendes). No specific permissions were required for the activities performed in the study area (Ipatinga, Minas Gerais state, Brazil).

### Phlebotomine sand fly fauna

Phlebotomine sand flies were captured using light traps operating from 6 pm to 8 am, during four consecutive days per month, from March 2015 to February 2016, in ten residences (one per district) placed inside and outside human dwellings, totaling twenty traps. The traps used are a modified version of the classical CDC light traps totally constructed using low cost materials [22]. All residences had favorable ecological conditions for the presence of phlebotomine sand flies, such as shaded areas, the presence of domestic animals and / or fruit trees [23–27], as well as a previous record of human and / or canine VL cases. Concerning favorable ecological conditions for the presence of phlebotomine sand flies, a study carried out in Belo Horizonte evaluated the potential risk factors for VL and showed that the presence of domestic animals, abundant organic matter, inadequate storage of garbage, stacked bricks and stones, trees and vegetable gardens in the households are related to its occurrence [26].

The phlebotomine sand flies were sorted from other insects and stored in hemolysis tubes containing 70% ethanol (males) or 6% dimethylsulfoxide (DMSO) (females). Subsequently, males and females were taken to the Laboratory of Leishmaniases of Instituto René Rachou for identification. The males were submitted to clarification by treatment with 10% KOH for 2 h, followed by 10% acetic acid for 20 min. After three washings with type I water for 15 min each, the samples were treated with a lactophenol solution composed of 100 ml of lactic acid, 200 ml of glycerin, 100 g of phenol and 100 ml of type I water. After the clarification process, the specimens were mounted. The females had the head and the last three abdominal segments dissected for specific identification. Berlese was used as the mounting medium in both cases. The remaining females were transferred to new tubes and individually frozen at -20°C for the study of natural *Leishmania* infection.

The captured phlebotomine sand flies of both sexes were identified using specific descriptions, taxonomic keys and comparison with specimens from the Reference Collection of Phlebotomine Sand flies of Instituto René Rachou. We adopted the species classification of phlebotomine sand flies proposed by Galati [24, 25, 28, 29].

The sum of the number of sand fly specimens captured during the three nights of capture in a given month was taken as representative of that month.

### Climate data

In order to establish a correlation between seasonality, climate conditions and the sand fly population, average monthly maximum temperature (°C), total rainfall (mm), and relative humidity (%) data were sourced from the nearest meteorological station to Ipatinga (station A554, OMM code 86802), with geographic coordinates of 19° 73' 57" S and 42° 13' 17" W. This station is located about 100 km away from the study area, in the city of Caratinga (Minas Gerais state, Brazil). Correlation analysis of Spearman (with a significance level of 0.05) was calculated using R software [30] to evaluate the influence of climate variables on the whole population density of phlebotomine sand flies captured per month. Average values for maximum temperature (°C) and total rainfall (mm) were calculated for the studied year and used to calculate the monthly local weather, in comparative terms. The months with temperatures above the yearly mean value were considered to be warm. Similarly, a total monthly rainfall above the yearly mean was taken as rainy period.

### Detection of *Leishmania* DNA in the phlebotomine sand flies

Test samples containing one or more specimens of the same of phlebotomine sand fly species were prepared exclusively with the female body portions stored at –20°C. When more than one specimen (up to ten) of the same species were captured in the same household and in the same month, they were pooled to compose one test sample. Otherwise, individual specimens constituted one test sample. All the test samples contained a) only females (i.e. no non-vectorial males), and b) only females that could be unambiguously identified morphologically (i.e. no cryptic sibling species).

Total DNA from each test sample was extracted using a commercial kit (GE HealthCare). The presence of *Leishmania* DNA was analyzed by Nested PCR (LnPCR) directed to the SSUrRNA gene that amplifies a conserved fragment for *Leishmania* [31,32]. All amplifications were performed with Illustra Pure Taq Ready-to-go PCR Beads kit (GE HealthCare), and the products were analyzed by electrophoresis in agarose gel. DNA from *Leishmania infantum* (strain MHOM/BR/PP75) and sterile distilled water were used as positive and negative controls, respectively.

The PCR-amplified bands were extracted from the agarose gel using QIAquick extraction kit (QIAGEN) and sequenced in both directions using the automated sequencer ABI3730 (Life Technologies). The consensus nucleotide sequences for each test sample were aligned and compared to databases of *Leishmania chagasi* (syn. *Leishmania infantum*, accession M81430), *Leishmania braziliensis* (accession M80292) and *Leishmania mexicana amazonensis* (accession M80293), using the Basic Local Aligned Search Tool (blast.ncbi.nlm.nih.gov).

The minimum rates of *Leishmania* infection (MIR) in the captured phlebotomine sand flies were calculated as the number of positive pools of a given phlebotomine sand fly species divided by the number of specimens of that species in the test sample and then multiplied by 100 [33].

### Canine visceral leishmaniasis

Canine VL diagnosis was performed using both active and passive methods. The canine survey aimed at diagnosing the disease in an active way, with trained public health agents visiting all the residences of the chosen district. In passive detection, the dog owner spontaneously took his animal to the Center of Zoonosis Control of the City Hall of Ipatinga for VL testing, between March 2015 and February 2016.

Canine VL diagnosis was performed according to the current protocol adopted by the Brazilian Ministry of Health: 1) Screening test: DPP rapid test–single use immunochromatographic test for detection of *Leishmania* specific antibodies; 2) Confirmation test: EIE–an immunoenzymatic assay for antibody detection using purified and soluble antigen of *Leishmania major*-like (strain MHOM/BR/71/BH121). The positive samples in the screening test were submitted to the confirmation test [34]. Both kits are produced by Biomanguinhos (Fiocruz) and are primarily destined to attend the demands of the Public Health Program of VL control and also to research projects developed by public institutions in Brazil. The protocol was performed by skilled professionals.

When the dog was serologically reactive in both tests, the owner’s residence was georeferenced. Subsequently the prevalence rates were plotted on a thematic map using the R software [30]. The canine population for each district corresponds to approximately 13.5% of the human population [35].

### Human visceral leishmaniasis

The data on human cases of VL per district of Ipatinga, during the study period, were obtained from the Epidemiological Surveillance Section of the Municipal Health Department of Ipatinga (provided by TS Mendes). The residential addresses were considered for the descriptive analysis of human cases, using tables, graphs and summary measures, such as averages, medians, proportions and rates. In order to analyze the spatial distribution of the human cases of VL, the Kernel density map was constructed and the incidence rates were plotted on a thematic map. These maps were performed using the “R” software [30].

### Ethics statement

This research was approved by the Animal Research Ethics Committee of Fundação Oswaldo Cruz (CEUA/Fiocruz) under number LW-16/15 (S1 Fig). All the procedures followed the technical norms established by the Federal Board of Veterinary Medicine (CFMV resolution no. 714/2002). Dog owners were informed of the project objectives and voluntarily signed a Free and Informed Consent Statement. The entomological captures were performed after voluntary signature of a Free and Informed Consent Statement by the residents. Data on human VL cases were kindly provided by the Epidemiological Surveillance Section of the City Health Department of Ipatinga. The field studies did not involve endangered or protected species.

## Results

### Phlebotomine sand fly fauna survey

The total numbers of phlebotomine sand flies captured in our study are compiled in Table 1, sorted by species and gender. About 53% of the phlebotomine sand flies were captured in the peridomicile area and 47% inside the residences (S1 Table).

**Table 1:** Phlebotomine sand fly species captured in the city of Ipatinga (Minas Gerais state, Brazil) using light traps, from March 2015 to February 2016.

Species	Females		Males		Males + Females	
	No.	%	No.	%	No.	%
*Brumptomyia avellari*	0	0.0	8	0.9	8	0.5
*Brumptomyia nitzulescui*	0	0.0	1	0.1	1	0.1
*Brumptomyia* spp.	7	1.1	0	0.0	7	0.5
*Evandromyia baculus*	1	0.2	0	0.0	1	0.1
*Evandromyia cortelezzii*	208	32.2	119	13.9	327	21.8
*Evandromyia lenti*	78	12.1	96	11.2	174	11.6
*Evandromyia termitophila*	1	0.2	0	0.0	1	0.1
*Lutzomyia longipalpis*	319	49.5	610	71.3	929	61.9
*Martinsmyia minasensis*	0	0.0	1	0.1	1	0.1
*Micropygomyia quinquefer*	3	0.5	4	0.5	7	0.5
*Nyssomyia intermedia*	11	1.7	7	0.8	18	1.2
*Nyssomyia whitmani*	8	1.2	6	0.7	14	0.9
*Pintomyia fischeri*	1	0.2	0	0.0	1	0.1
*Pintomyia pessoai*	1	0.2	0	0.0	1	0.1
*Pressatia choti*	1	0.2	2	0.2	3	0.2
*Sciopemyia sordellii*	5	0.8	2	0.2	7	0.5
*Trichopygomyia longispina*	1	0.2	0	0.0	1	0.1
Total	645	100.0	856	100.0	1501	100.0

**Fig 2:**
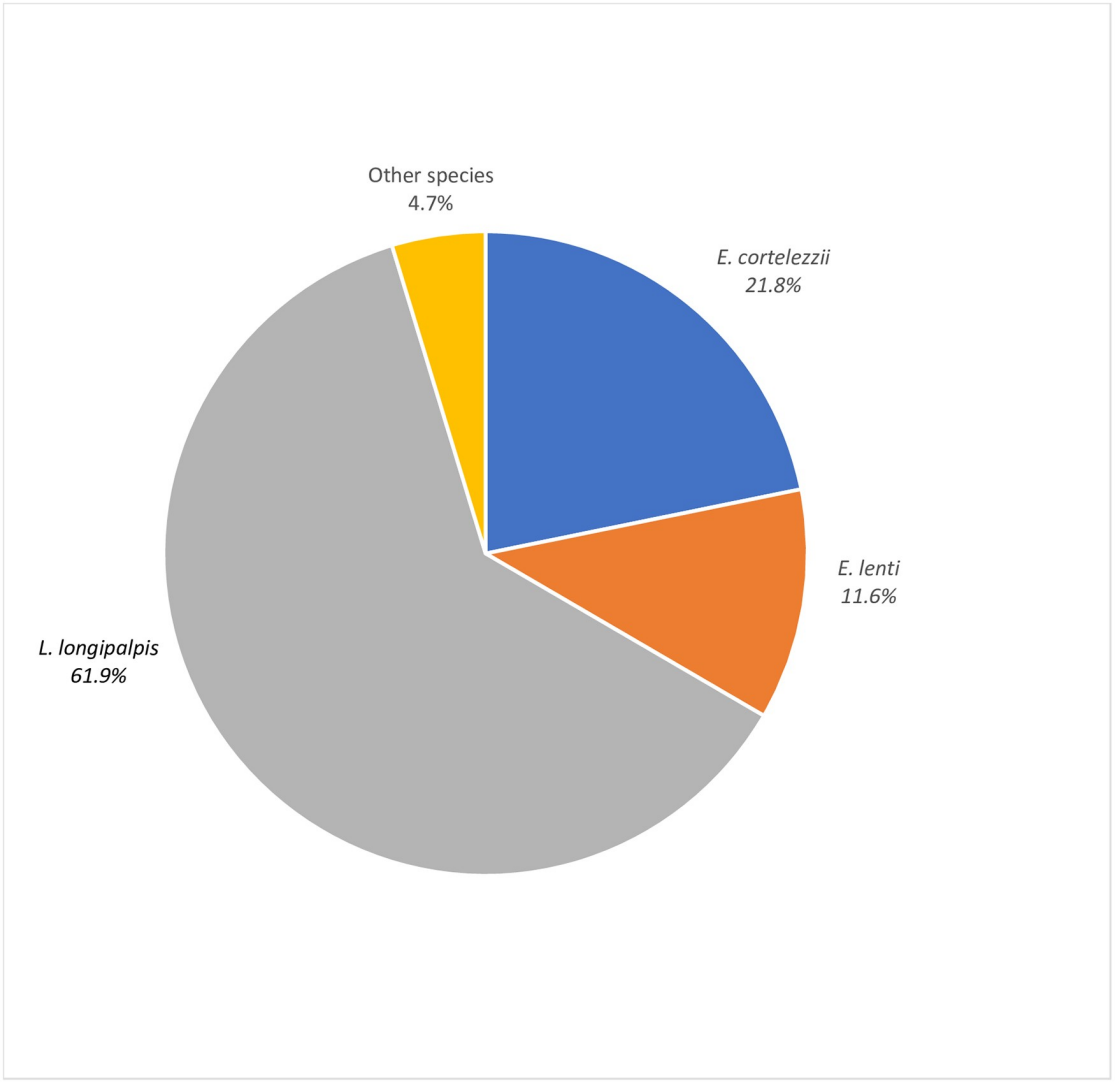
Species distribution of phlebotomine sand flies in the city of Ipatinga, (Minas Gerais, Brazil). Species with relative percentages lower or equal to 1.2% were grouped under “other species”. Study period: March 2015 to February 2016.

From a total of 1,501 captured specimens of phlebotomine sand flies, males were slightly predominant (57%) over females (43%). The predominant species, for both genders, were *Lutzomyia longipalpis* (61.9%), *Evandromyia cortelezz*ii (21.8%), and *Evandromyia lenti* (11.6%) with female/male ratios of 0.5, 1.7, and 0.8, respectively. Phlebotomine sand flies belonging to other species accounted for 4.7% of the total captured specimens (Fig 2). Relative percentages of females belonging to the three predominant species followed the same decreasing order observed for both genders: 49.5% for *L*. *longipalpis*, 32.2% for *E*. *cortelezzii*, and 12.1% for *E*. *lenti*. The species *Nyssomyia whitmani* and *Nyssomyia intermedia*, both species of biological importance, reached about 1% each of the total number of phlebotomine sand flies.

**Fig 3:**
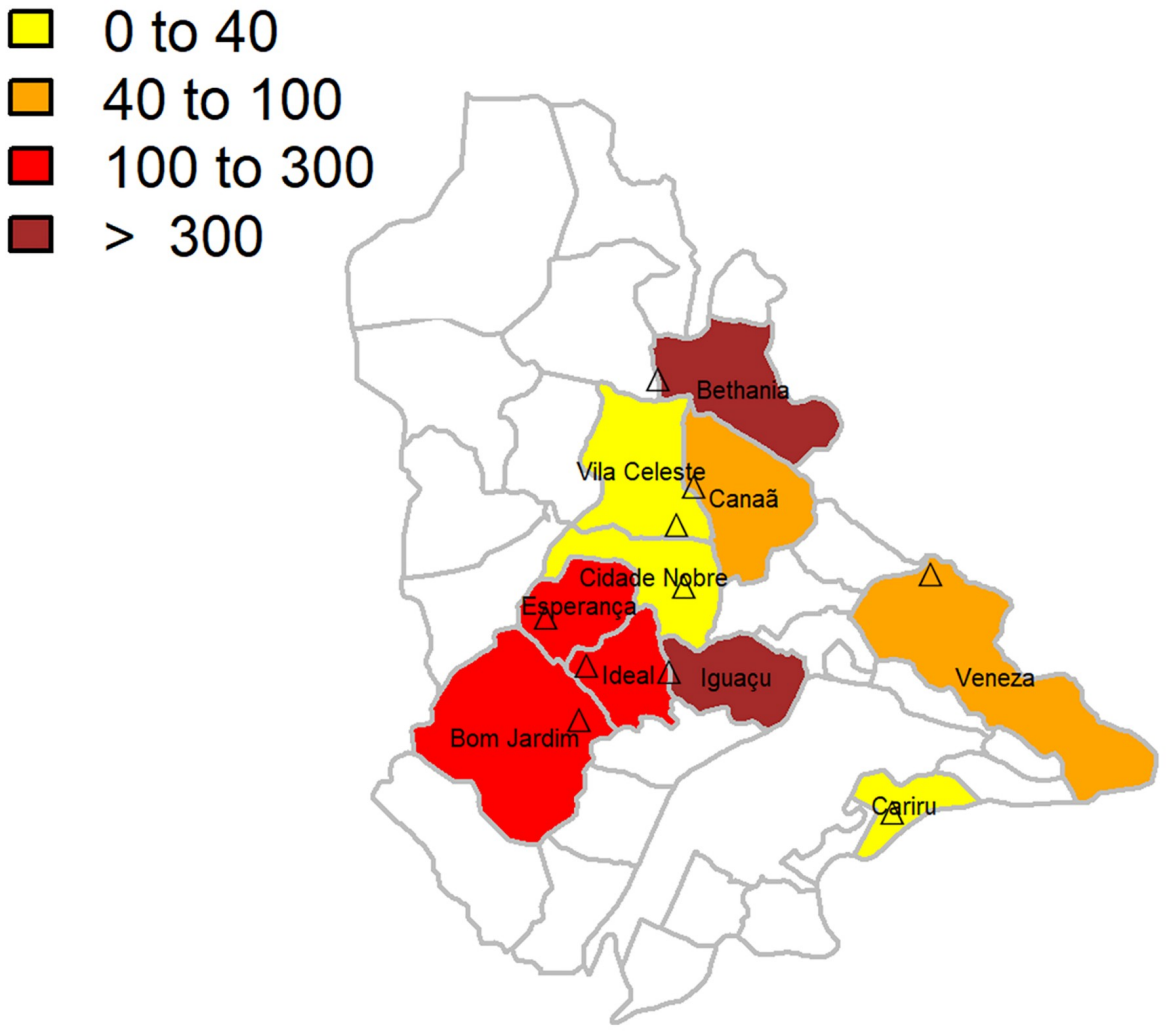
Population density of phlebotomine sand flies captured per district in the city of Ipatinga, Minas Gerais, Brazil. Color background represents total phlebotomine sand flies captured per district, throughout one year. Districts shaded in white were not selected for the study. The entomological capture sites are indicated by triangles. District names are in black font. Study period: March 2015 to February 2016.

The spatial distribution of the entomological capture points in the districts of Ipatinga is shown in Fig 3. In eight out of the ten districts selected for the study, *L*. *longipalpis* was the predominant species throughout the year: Bethânia, Bom Jardim, Canaã, Cidade Nobre, Iguaçu, Esperança, Ideal and Veneza. The species *E*. *cortelezzii* was prevalent in the district of Vila Celeste, and *N*. *intermedia* in the district of Cariru (S2 Table). The districts with the higher population density (>300) of phlebotomine sand flies, considering all species, were Bethânia and Iguaçu (Fig 3).

**Fig 4:**
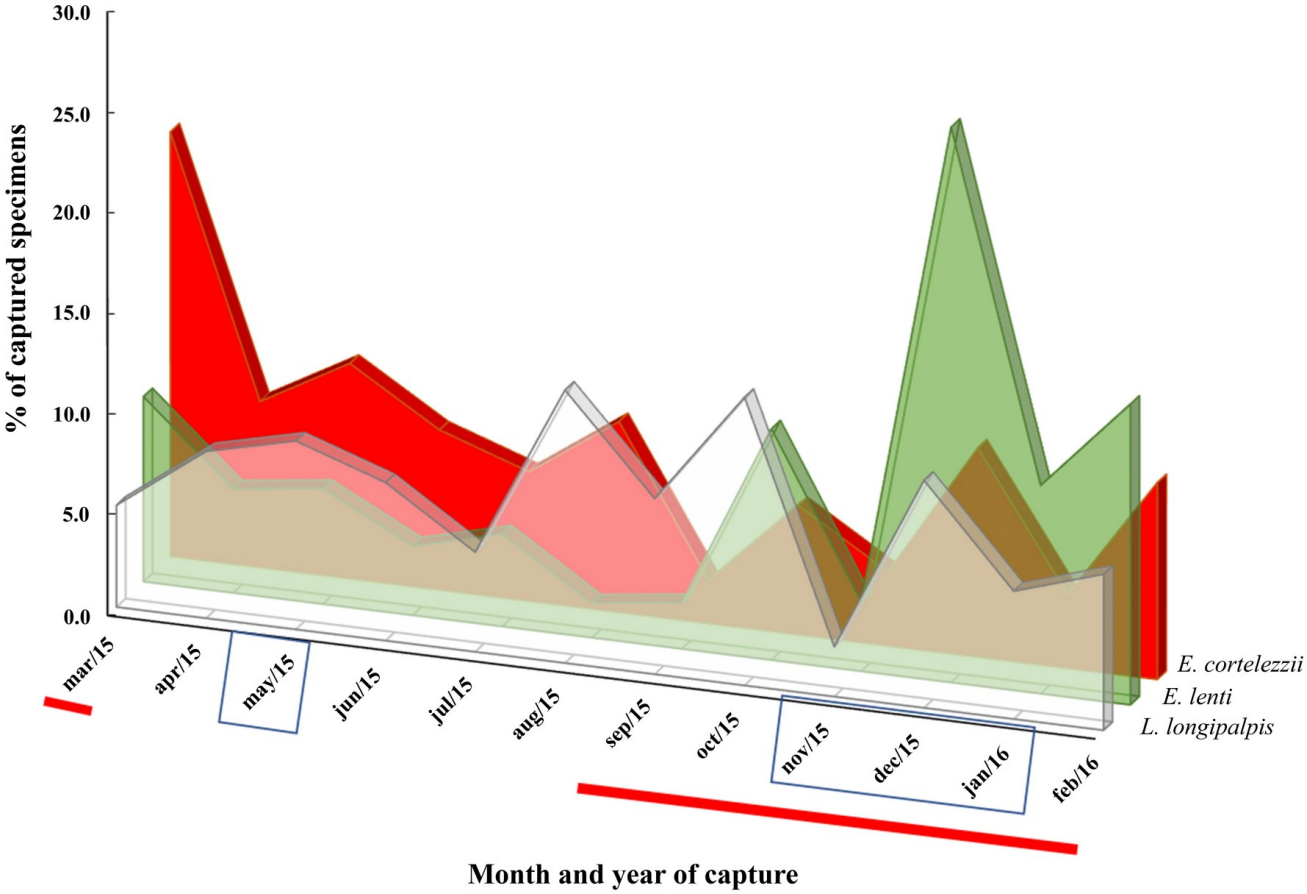
Monthly percentages of the three predominant species of phlebotomine sand flies captured in Ipatinga, Minas Gerais (Brazil). The total annual number of each species was taken as 100%. Color legend: *E*. *cortelezzii* in red, *E*. *lenti* in green, and *L*. *longipalpis* in transparent gray. Rainy months (with precipitation index higher than the mean calculated for the one-year period) are inside rectangles. The red line below the X-axis indicates warmer months with temperatures above the mean temperature calculated for the year. Period of study: March 2015 to February 2016.

In order to evaluate whether climate variables (temperature, relative air humidity and rainfall) had any effects on the population density of phlebotomine sand flies, we first investigated any possible associations between each pair of climate variables themselves. No significant association was found, except for a tendency of concomitant increase of relative air humidity with rainfall, through the months. Similarly, there was no statistically significant association between the monthly number of phlebotomine sand flies and any of the climate variables. The climate variables did not interfere with the monthly number of *L*. *longipalpis* either. The monthly number of specimens captured for the three predominant species throughout the year is expressed in Fig 4. The species *E*. *cortelezzii* showed an increased percentage (21.1%) in March (warm and dry weather conditions). On the other hand, *E*. *lenti* displayed a peak (27.6%) in December, which was also warm and rainy. *L*. *longipalpis* population was more homogeneously distributed over the year of capture, with less pronounced populational peaks in August, October and December, in comparison with *E*. *cortelezzii* and *E*. *lenti*.

### *Leishmania* DNA in phlebotomine sand flies

**Fig 5:**
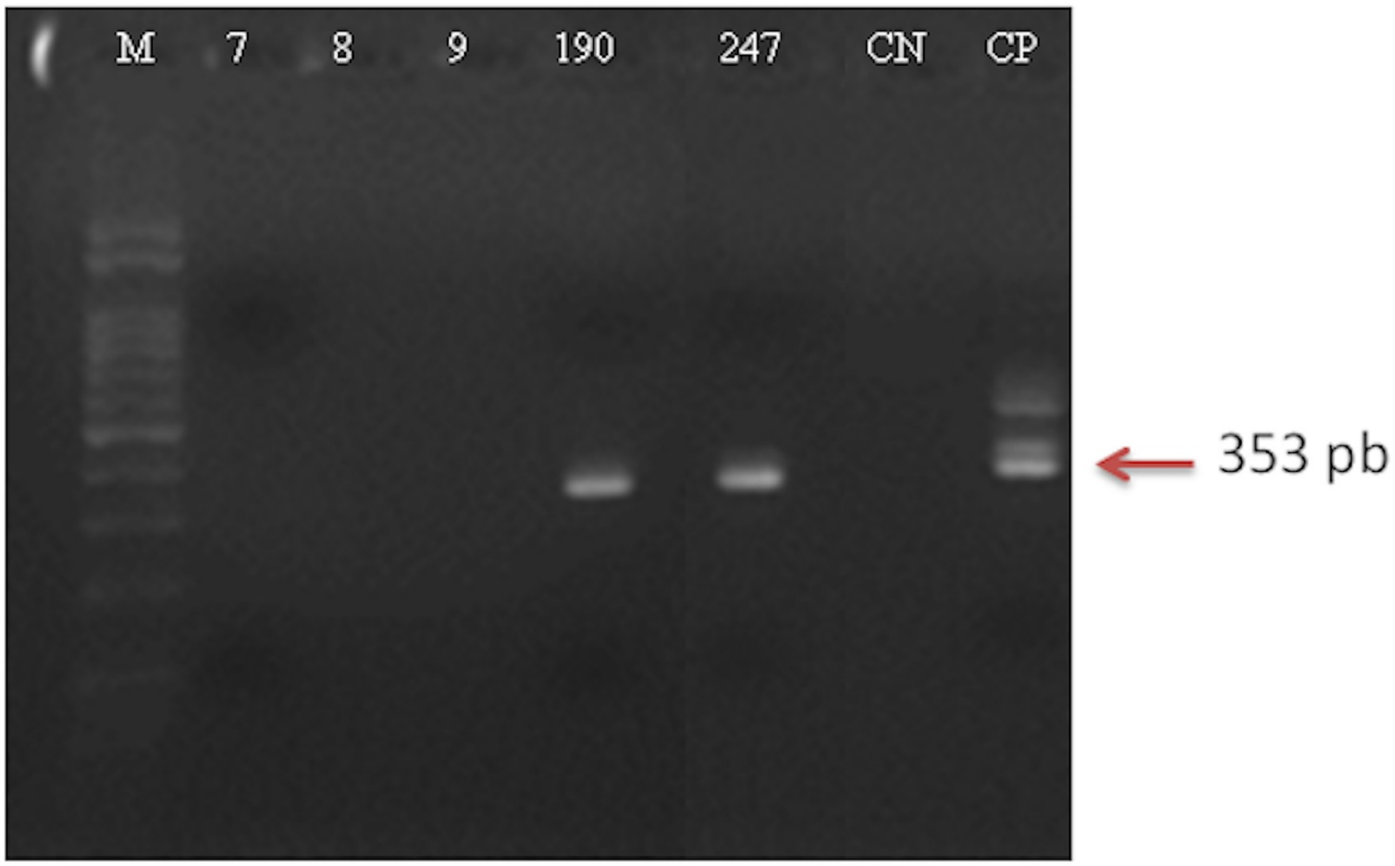
Electrophoresis of LnPCR amplification products of total phlebotomine sand fly DNA on 2% agarose stained by ethidium bromide. A 353bp PCR product is evidence of the presence of *Leishmania* DNA. Sample wells: M- 100pb ladder; 7, 8 and 9- Representative negative test samples for *Leishmania* DNA; 190—Positive test sample for *Leishmania* DNA in *L*. *longipalpis*; 247- Positive test sample for *Leishmania* DNA in *E*. *cortelezzii*. CN- Negative control (without DNA); CP- Positive control: *Leishmania infantum* (strain MHOM/BR/PP75).

The presence of *Leishmania* DNA was investigated in 285 test samples of total DNA from the female phlebotomine sand flies captured in our study (Table 2). Among them, the 353 bp fragment characteristic of the *Leishmania* genus was observed in one test sample of *L*. *longipalpis* (numbered 190) and in one test sample of *E*. *cortelezzii* (numbered 247), after LnPCR (Fig 5).

**Table 2:** Natural presence of *Leishmania* DNA in total DNA extracted exclusively from females of captured phlebotomine sand flies. When more than one specimen (up to ten) of the same species was captured in the same household and in the same month, they were pooled as one test sample. Otherwise, individual specimens constituted one test sample.

Species	No. females captured	No. of test samples	
		Analyzed	Positive for *Leishmania* DNA
*Brumptomyia* spp.	7	7	0
*E*. *baculus*	1	1	0
*P*. *choti*	1	1	0
*E*. *cortelezzii*	208	93	1
*P*. *fischeri*	1	1	0
*N*. *intermedia*	11	11	0
*E*. *lenti*	78	38	0
*L*. *longipalpis*	319	115	1
*T*. *longispina*	1	1	0
*M*. *minasensis*	0	0	0
*P*. *pessoai*	1	1	0
*M*. *quinquefer*	3	3	0
*S*. *sordellii*	5	4	0
*E*. *termitophila*	1	1	0
*N*. *whitmani*	8	8	0
**Total**	**645**	**285**	**2**

**Fig 6:**
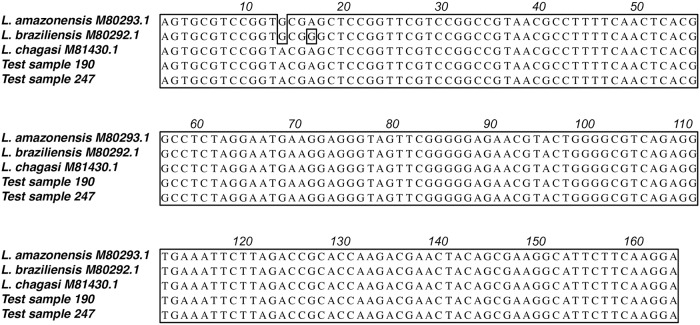
Multiple nucleotide alignment of the characteristic *Leishmania* DNA fragment after LnPCR using total phlebotomine sand fly DNA as template. The nucleotide mutations that characterize each *Leishmania* species are shown in the first line. *Leishmania* sequences used as references are those deposited in the GenBank [30].

Further nucleotide sequencing of the amplified fragments in the two positive test samples for *Leishmania* indicated the presence of *Leishmania infantum* DNA in both cases (Fig 6). The minimum rates of natural infection (MIR) by *Leishmania infantum* were estimated as 0.3% for *L*. *longipalpis* and 0.5% for *E*. *cortelezzii*. In the first case, the test sample was a pool of seven specimens, whereas the latter contained a single specimen of *E*. *cortelezzii*.

### Canine visceral leishmaniasis

A total of 9136 blood samples were obtained in the canine serological survey, of which 4183 (45.8%) were actively collected and 4953 (54.2%) originated from passive survey. Canine VL was serologically confirmed in 1355 (14.8%) dogs (Table 3).

**Table 3:** Canine visceral leishmaniasis in the city of Ipatinga, Minas Gerais State, Brazil. Data were obtained from active and passive surveys from March 2015 to February 2016. Dogs with positive DPP (screening test) and ELISA (confirmatory test) results were considered serologically positive for VL, in accordance with the current protocol adopted by the Brazilian Ministry of Health for canine VL diagnosis [34].

District	Number of dogs tested	Number of positive dogs	
		DPP	DPP and ELISA
Bethânia	2202	737	410
Canaã	2364	690	309
Cidade Nobre	455	133	55
Bom Jardim	857	298	128
Esperança	615	239	94
Ideal	313	114	49
Iguaçu	285	105	42
Veneza	306	115	43
Vila Celeste	658	287	113
Cariru	41	8	1
**Subtotal**	**8096**	**2726**	**1244**
Others	1040	353	111
**Total**	**9136**	**3079**	**1355**

**Fig 7:**
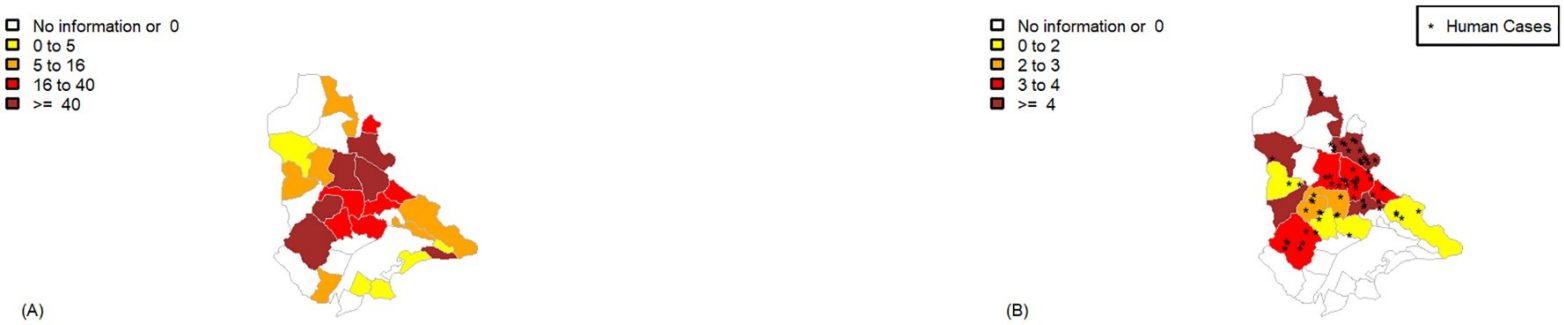
Distribution of canine and human VL in the districts of Ipatinga (Minas Gerais state, Brazil). A. Prevalence rate (per 1,000) of canine VL cases; B. Incidence rate (per 10,000) of human VL cases. Human cases notified to the local Department of Health are indicated by “*” on the corresponding district on the map. The entomological capture sites are indicated by green diamonds. Period: March 2015 to February 2016.

Fig 7 shows the distribution of canine and human VL infection in the districts of Ipatinga and the entomological capture sites in the study period. The prevalence rates of canine VL based on the estimated evenly-distributed dog population considerably varied among the studied districts. Bethânia, Canaã, Vila Celeste, Esperança and Bom Jardim had higher prevalence rates (Fig 7A). The district of Cariru did not show many canine cases but, given the population size of the district, the number of canine VL cases was proportionally high.

### Human cases of VL

The districts with the higher VL incidence were Bethânia and Canaã, with eighteen and nine cases, respectively. In most studied districts, higher prevalences of canine VL overlapped with higher human VL incidences (Fig 7B). The Bethânia district appears in the highest rates category in both maps.

In the same period, 64 cases of VL were registered by the Epidemiological Surveillance Section of the Municipal Department of Health of Ipatinga: 36 (56.2%) were men and 28 (43.8%) were women. Approximately 32% of the cases were 50 to 89 years old and 25% were children up to 5 years of age. Ten deaths due to VL were registered in the same period, seven of which occurred in the districts under study (data provided by TS Mendes).

**Fig 8:**
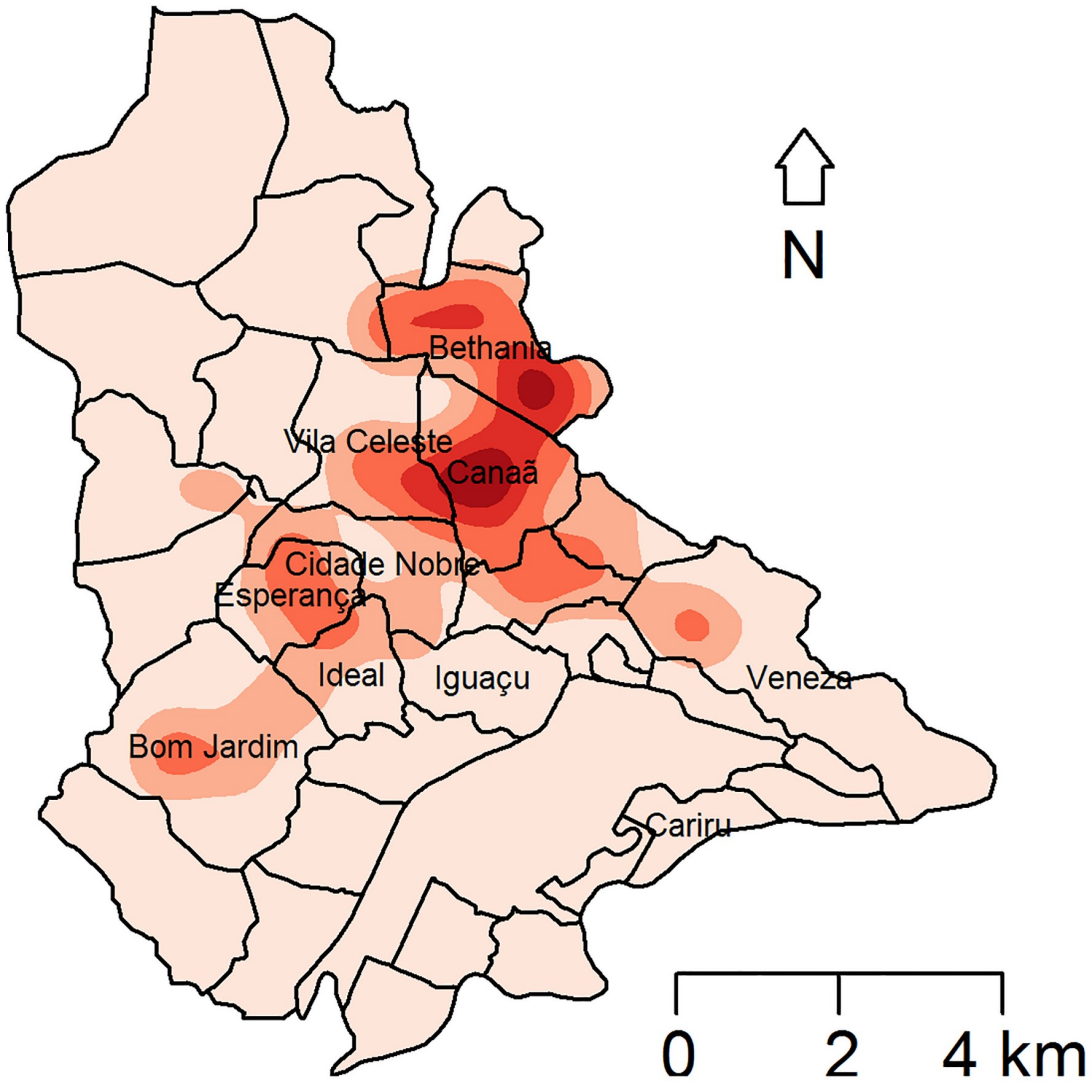
Kernel density map showing the distribution of human visceral leishmaniasis in Ipatinga (Minas Gerais state, Brazil). Red spots represent the areas with the highest numbers of human VL cases. Study period: March 2015 to February 2016.

The Kernel density map (Fig 8) shows main hotspots in the districts of Bethânia and Canaã. However, VL was widely distributed in nine of the ten studied districts.

## Discussion

In the present study we describe, for the first time, important aspects of the transmission chain of VL in area of recent transmission but with high epidemiological transmission risk of the disease in humans. We investigated the entomological fauna, the prevalence of canine VL and the reported cases of human VL in Ipatinga, an economically important city for the state of Minas Gerais and Brazil. Our results were compiled in thematic maps indicating the actual situation of VL in the city.

The importance of analyzing the population density of vectors and correlating it with aspects related to the peridomicile is extensively discussed in the literature [23, 28, 36, 37]. This relationship is directly associated with the degree of adaptability of domestic animals and the vectors’ attractiveness to the environmental conditions, such as: 1. abundance of organic matter favoring the maturation of immature forms; 2. large number of domestic animals as a blood source for hematophagous female adults; 3. a variety of microhabitats that protect them from adverse conditions [28, 36, 37]. Taking these factors into account, the entomological traps were strategically mounted in shadowed areas and near vegetation, whenever possible. Many residences had fruit trees and/or domestic animals (dogs or chickens) in its peridomicile that may have favored the diverse fauna of captured phlebotomine sand flies captured in our study. The availability of organic matter in the soil and the presence of chickens may increase potential risk factors for the occurrence of VL [23, 26].

In Brazil, *L*. *longipalpis* is the main phlebotomine sand fly species responsible for VL transmission, followed by *Lutzomyia cruzi*, which is more important in the states of Mato Grosso and Mato Grosso do Sul [38–41]. Due to its high vector capacity, anthropophilia and prevalence in the intra and peridomicile areas, *L*. *longipalpis* is considered the species of major medical importance in VL transmission [42, 43]. *L*. *longipalpis* was the predominant species (61.9%) captured in Ipatinga, corroborating other studies carried out in known endemic areas of VL in Minas Gerais State [6, 44–48]. *L*. *longipalpis* specimens were present every month and in every district under study. *E*. *cortelezzii* and *E*. *lenti* were also captured in major amounts.

Since climate factors, such as temperature, air humidity and rainfall, variably interfere with the population density of phlebotomine sand flies [49], we analyzed the local seasonal profile of the three predominant species in our study: *L*. *longipalpis*, *E*. *cortelezzii* and *E*. *lenti*. Although it has been generally observed that the population density of phlebotomine sand flies generally increases in warm and humid months and decreases in dry and cold months, each of the three major species displayed a different seasonal profile [6, 44, 45, 50]. No statistical association could be found between any climate factor and the population density of any of these species.

*L*. *infantum* DNA was detected in *L*. *longipalpis* and *E*. *cortelezzii* with estimated MIRs of 0.3% and 05%, respectively. In nature, even in endemic and high-VL transmission areas, the prevalence of *Leishmania* DNA in the total population of phlebotomine sand flies is low [51]. Therefore, our estimated values are compatible with other studies described in the literature that found a value between 0.16% and 1.25% [51–54]. The presence of *L*. *infantum* DNA in *E*. *cortelezzii* was described before [55–57]. Rosa *et al*. [58] raised the possibility that this phlebotomine sand fly could be participating in VL transmission, since they found specimens naturally infected by *Leishmania* in endemic areas of VL. However, a number of criteria must be attended in order to consider a given species of phlebotomine sand fly a competent vector of leishmaniases: a geographical distribution coincident with that of the disease, high density of the species, detection of natural infection by the parasite, its transmission capacity and food preference [59]. The whole set of data available so far is insufficient to conclude anything about the actual epidemiological role of *E*. *cortelezzii*, if any, in VL transmission. Further studies are needed in order to specifically investigate this point.

The species *N*. *intermedia* and *N*. *whitmani* were captured in our entomological fauna survey, similarly to surveys conducted in other endemic areas of TL in Minas Gerais, but which did not provide data about their natural infection by *Leishmania* [50, 60, 61]. Ipatinga is an endemic area of TL with 191 cases reported between 2009 and 2015 [17]. In Cariru alone, one of the districts included in the present study, fourteen cases of TL (36%) were reported of the total of 39 for the whole city between March 2015 and February 2016 (data provided by TS Mendes). Remarkably, in this district, the predominant species of phlebotomine sand fly captured was *N*. *intermedia*, vector of TL. Unfortunately, up to the present time, there are no studies on the etiological agent of TL. *L*. *braziliensis* is the most frequent infecting parasite in endemic TL regions. But, despite the abundance of *N*. *intermedia* and *N*. *whitmani*, we did not detect *L*. *braziliensis* DNA in any of the test samples analyzed. Some works in the literature suggest that *L*. *infantum* could be an etiological agent of TL. Lara-Silva et al [62] detected *L*. *infantum* DNA in *Rattus norvegicus*, which is known to participate in the wild transmission cycle of the disease [63, 64]. Although rare, *L*. *infantum* has been found in patients with cutaneous lesions. Oliveira-Neto et al. [65] described a first case of cutaneous lesions induced by a “viscerotropic” *Leishmania*. At that time, however, the authors called the attention to the fact that assumptions about the etiological agent based solely on clinical examination and epidemiological data might led to erroneous conclusions. Later, Lyra et al. [66] reported the first case of TL with exclusive cutaneous lesions in Rio de Janeiro (Brazil) and concluded that *L*. *infantum* was the etiological agent there. Unfortunately, our data do not allow any conclusion about that and further studies are needed on endemic regions of TL.

The use of geoprocessing in epidemiological studies has contributed to improve the description of diseases in relation to spatially distributed variables. Spatial analysis techniques have been largely used in the study of leishmaniases, allowing the analysis of spatial dependence between canine and human VL, vector distribution, and characterization of areas with high incidence and risk of death [67–69]. The density of infected dog population was positively correlated with the relative risk of contracting human VL [70]. A correlation between seropositive dogs and human cases of VL was described in Belo Horizonte, the capital of Minas Gerais State [67]. In our study, the thematic maps suggested an association between the canine prevalence and human incidence on a visual basis. Spatial analysis allowed the visualization of regions with the highest VL prevalence, therefore enabling the identification of target areas and risk factors in order to guide more effective strategies of control / prevention. Among these are the removal of serologically positive dogs and insecticidal spraying of the households in months with a higher population density of vectors.

In practical terms, the Zoonosis Control Center of Ipatinga, in accordance with the procedures determined by the Brazilian Ministry of Health, only adopts chemical control measures against the vectors in areas with notified autochthonous human VL, and after entomological investigation [15]. As for the domestic reservoir, the presence of asymptomatic dogs for VL is a difficulty faced by the public health organs. Although these dogs present no clinical sign of the disease, some of them are infective for *Leishmania* and may act as a source of the parasite [71]. This reinforces that the removal of seropositive dogs, although controversial, is one important measure to control human VL [10, 72–75]. In addition, Oliveira [67] suggested that every VL case should be monitored for at least six months, since the occurrence of a human case in one household increases the chance of observing another human case in the same house within the next three months but does not change the chances of another event in the district.

Ipatinga is, today, the municipality with the highest human population density in the Steel Valley and the one that most suffers from the occurrence of VL. It has a Zoonoses Control Center and invests in VL control actions in accordance with to the PSCVL of the Brazilian Ministry of Health. The actions are performed according to the existing epidemiological reality and are prioritized to areas of greater occurrence of human and canine cases of VL. Based on the presence of *L*. *longipalpis* carrying *L*. *infantum* DNA, the prevalence of canine and human VL cases, we can conclude that the transmission cycle of VL is active in Ipatinga. Although only symptomatic human cases were included in our analysis, the spatial techniques used here allowed us to visualize a positive relationship between canine and human cases of VL and also to perceive the areas with the highest priority for application of control measures against the disease in Ipatinga.

According to the manual of the PSCVL of the Ministry of Health, the main objective of the Program is the reduction in the number of cases and number of deaths, since the percentage of death due to VL can reach over 90% if the patients are left untreated. Besides efforts aiming at early diagnosis and treatment of the disease, the reduction of risk transmission through the control of reservoir and vectors is another important point of the PSCVL. The objective of our study was to contribute to the comprehension of the local epidemiological profile in Ipatinga concerning vectors and reservoirs of VL.

## Supporting information

S1 Fig License for scientific use of animals from the Ethics Committee on the use of animals (CEUA/Fiocruz LW-16/15). (PDF)

S1 Table Phlebotomine sand flies captured per species, gender, and peri or intradomicile light traps in the city of Ipatinga, State of Minas Gerais (Brazil). (DOCX)

S2 Table Phlebotomine sand fly species captured per district of Ipatinga, State of Minas Gerais (Brazil). (DOCX)
