# Supplementary material for: Ecoepidemiological aspects of visceral leishmaniasis in an endemic area in the Steel Valley in Brazil: An ecological approach with spatial analysis
Source: PLoS One. 2018 Oct 30;13(10):e0206452. doi: 10.1371/journal.pone.0206452 (PMC6207327; doi:10.1371/journal.pone.0206452)
Supplement: S1 Fig — (PDF) [file pone.0206452.s001.pdf]

Ministério da Saúde

FIOCRUZ
Fundação Oswaldo Cruz

Vice-presidência de Pesquisa e Laboratórios de Referência

CEUA Fiocruz

Comissão de Ética no Uso de Animais

LICENÇA

LW-16/15

Certificamos que o protocolo (P-81/14-2), intitulado "EPIDEMIOLOGIA E CONTROLE DA LEISHMANIOSE VISCERAL NO MUNICÍPIO DE IPATINGA, REGIÃO METROPOLITANA DO VALE DO AÇO, ESTADO DE MINAS GERAIS, BRASIL.", sob a responsabilidade de EDELBERTO SANTOS DIAS, atende ao disposto na Lei 11794/08, que dispõe sobre o uso científico no uso de animais, inclusive aos princípios da Sociedade Brasileira de Ciência em Animais de Laboratório (SBCAL). A referida licença não exime a observância das Leis e demais exigências legais na vasta legislação nacional.

Esta licença tem validade até 30/03/2018 e inclui o uso total de :

Canis familiaris
- **50 Machos.**
- **50 Fêmeas.**

Rio de Janeiro, 30 de março de

Octavio Augusto França Presgrave
Coordenador da CEUA

Octavio A. F. Presgrave
Coordenador
CEUA/FIOCRUZ
SIAPE 04626550
30/03/15

Comissão de Ética no Uso de Animais
Vice-presidência de Pesquisa e Laboratórios de Referência - Fundação Oswaldo Cruz
Av. Brasil, 4036 - Prédio da Expansão - sala 200 - Manguinhos - Rio de Janeiro / RJ
Telefone: (21) 3882.9121 e-mail: ceua@fiocruz.br
