# Supplementary material for: Ecoepidemiological aspects of visceral leishmaniasis in an endemic area in the Steel Valley in Brazil: An ecological approach with spatial analysis
Source: PLoS One. 2018 Oct 30;13(10):e0206452. doi: 10.1371/journal.pone.0206452 (PMC6207327; doi:10.1371/journal.pone.0206452)
Supplement: S1 Table — (DOCX) [file pone.0206452.s002.docx]

**S2 Table. Phlebotomine sandflies captured per species, gender, and peri or intradomicile light traps**. The study was performed in Ipatinga, State of Minas Gerais (Brazil) from March 2015 to February 2016.

| **Species / Gender** | **Male** | | **Female** | | **TOTAL** | **%** |
| --- | --- | --- | --- | --- | --- | --- |
|  |  |  |  |  |  |  |
|  | **PERI** | **INTRA** | **PERI** | **INTRA** |  |  |
| *Brumptomyia avellari* | 2 | 6 | 0 | 0 | 8 | 0.5 |
| *Brumptomyia nitzulescui* | 1 | 0 | 0 | 0 | 1 | 0.1 |
| *Brumptomyia* spp. | 0 | 0 | 3 | 4 | 7 | 0.5 |
| *Evandromyia baculus* | 0 | 0 | 0 | 1 | 1 | 0.1 |
| *Pressatia choti* | 1 | 1 | 0 | 1 | 3 | 0.2 |
| *Evandromyia cortelezzii* | 45 | 74 | 77 | 131 | 327 | 21.8 |
| *Pintomyia fischeri* | 0 | 0 | 0 | 1 | 1 | 0.1 |
| *Nyssomyia intermedia* | 5 | 2 | 4 | 7 | 18 | 1.2 |
| *Evandromyia lenti* | 59 | 37 | 38 | 40 | 174 | 11.6 |
| *Lutzomyia longipalpis* | 398 | 212 | 149 | 170 | 929 | 61.9 |
| *Trichopygomyia longispina* | 0 | 0 | 0 | 1 | 1 | 0.1 |
| *Martinsmyia minasensis* | 0 | 1 | 0 | 0 | 1 | 0.1 |
| *Pintomyia pessoai* | 0 | 0 | 0 | 1 | 1 | 0.1 |
| *Micropygomyia quinquefer* | 1 | 3 | 1 | 2 | 7 | 0.5 |
| *Sciopemyia sordellii* | 1 | 1 | 5 | 0 | 7 | 0.5 |
| *Evandromyia termitophila* | 0 | 0 | 0 | 1 | 1 | 0.1 |
| *Nyssomyia whitmani* | 2 | 4 | 4 | 4 | 14 | 0,9 |
| **TOTAL** | **515** | **341** | **281** | **364** | **1501** | **100%** |
