# Supplementary material for: Ecoepidemiological aspects of visceral leishmaniasis in an endemic area in the Steel Valley in Brazil: An ecological approach with spatial analysis
Source: PLoS One. 2018 Oct 30;13(10):e0206452. doi: 10.1371/journal.pone.0206452 (PMC6207327; doi:10.1371/journal.pone.0206452)
Supplement: S2 Table — (DOCX) [file pone.0206452.s003.docx]

**S3 Table. Phlebotomine sandfly species captured per district of Ipatinga, State of Minas Gerais (Brazil).** Period of study: March 2015 to February 2016.

|  | **Vila Celeste** | **Canaã** | **Bethânia** | **Veneza** | **Cariru** | **Iguaçu** | **Cidade Nobre** | **Bom Jardim** | **Esperança** | **Ideal** | **TOTAL** |  |
| --- | --- | --- | --- | --- | --- | --- | --- | --- | --- | --- | --- | --- |
| **Species / District** |  |  |  |  |  |  |  |  |  |  |  | **%** |
|  |  |  |  |  |  |  |  |  |  |  |  |  |
| *Brumptomyia avellari* | 0 | 0 | 7 | 0 | 1 | 0 | 0 | 0 | 0 | 0 | 8 | 0.5 |
| *Brumptomyia nitzulescui* | 0 | 0 | 1 | 0 | 0 | 0 | 0 | 0 | 0 | 0 | 1 | 0.1 |
| *Brumptomyia* spp*.* | 0 | 0 | 4 | 0 | 2 | 0 | 0 | 1 | 0 | 0 | 7 | 0.5 |
| *Evandromyia baculus* | 0 | 0 | 1 | 0 | 0 | 0 | 0 | 0 | 0 | 0 | 1 | 0.1 |
| *Pressatia choti* | 0 | 0 | 0 | 0 | 3 | 0 | 0 | 0 | 0 | 0 | 3 | 0.2 |
| *Evandromyia cortelezzii* | 22 | 32 | 89 | 18 | 0 | 74 | 3 | 54 | 14 | 21 | 327 | 21.8 |
| *Pintomyia fischeri* | 0 | 0 | 0 | 0 | 1 | 0 | 0 | 0 | 0 | 0 | 1 | 0.1 |
| *Nyssomyia intermedia* | 3 | 0 | 2 | 2 | 11 | 0 | 0 | 0 | 0 | 0 | 18 | 1.2 |
| *Evandromyia lenti* | 0 | 5 | 62 | 5 | 2 | 65 | 0 | 30 | 1 | 4 | 174 | 11.6 |
| *Lutzomyia longipalpis* | 11 | 40 | 150 | 22 | 1 | 224 | 34 | 154 | 185 | 108 | 929 | 61.9 |
| *Trichopygomyia longispina* | 0 | 0 | 1 | 0 | 0 | 0 | 0 | 0 | 0 | 0 | 1 | 0.1 |
| *Martinsmyia minasensis* | 0 | 0 | 0 | 0 | 0 | 0 | 0 | 1 | 0 | 0 | 1 | 0.1 |
| *Pintomyia pessoai* | 0 | 0 | 0 | 0 | 1 | 0 | 0 | 0 | 0 | 0 | 1 | 0.1 |
| *Micropygomyia quinquefer* | 0 | 0 | 0 | 0 | 0 | 0 | 0 | 7 | 0 | 0 | 7 | 0.5 |
| *Sciopemyia sordellii* | 0 | 0 | 0 | 0 | 0 | 3 | 0 | 3 | 1 | 0 | 7 | 0.5 |
| *Evandromyia termitophila* | 0 | 0 | 1 | 0 | 0 | 0 | 0 | 0 | 0 | 0 | 1 | 0.1 |
| *Nyssomyia whitmani* | 0 | 0 | 4 | 0 | 0 | 0 | 0 | 8 | 1 | 1 | 14 | 0.9 |
| **TOTAL** | **36** | **77** | **322** | **47** | **22** | **366** | **37** | **258** | **202** | **134** | **1501** | **100** |
